# Increasing the metabolic capacity of *Escherichia coli* for hydrogen production through heterologous expression of the *Ralstonia eutropha* SH operon

**DOI:** 10.1186/1754-6834-6-122

**Published:** 2013-08-26

**Authors:** Dipankar Ghosh, Ariane Bisaillon, Patrick C Hallenbeck

**Affiliations:** 1 Département de Microbiologie et Immunologie, Université de Montréal, CP 6128 Succursale Centre-ville, Montréal, Québec, Canada H3C 3J7

**Keywords:** Biohydrogen, Metabolic engineering, Heterologous expression, Hydrogen production from NADH

## Abstract

**Background:**

Fermentative hydrogen production is an attractive means for the sustainable production of this future energy carrier but is hampered by low yields. One possible solution is to create, using metabolic engineering, strains which can bypass the normal metabolic limits to substrate conversion to hydrogen. *Escherichia coli* can degrade a variety of sugars to hydrogen but can only convert electrons available at the pyruvate node to hydrogen, and is unable to use the electrons available in NADH generated during glycolysis.

**Results:**

Here, the heterologous expression of the soluble [NiFe] hydrogenase from *Ralstonia eutropha* H16 (the SH hydrogenase) was used to demonstrate the introduction of a pathway capable of deriving substantial hydrogen from the NADH generated by fermentation. Successful expression was demonstrated by in vitro assay of enzyme activity. Moreover, expression of SH restored anaerobic growth on glucose to *adhE* strains, normally blocked for growth due to the inability to re-oxidize NADH. Measurement of in vivo hydrogen production showed that several metabolically engineered strains were capable of using the SH hydrogenase to derive 2 mol H_2_ per mol of glucose consumed, close to the theoretical maximum.

**Conclusion:**

Previous introduction of heterologous [NiFe] hydrogenase in *E. coli* led to NAD(P)H dependent activity, but hydrogen production levels were very low. Here we have shown for the first time substantial in vivo hydrogen production by a heterologously expressed [NiFe] hydrogenase, the soluble NAD-dependent H_2_ase of *R. eutropha* (SH hydrogenase). This hydrogenase was able to couple metabolically generated NADH to hydrogen production, thus rescuing an alcohol dehydrogenase (*adhE*) mutant. This enlarges the range of metabolism available for hydrogen production, thus potentially opening the door to the creation of greatly improved hydrogen production. Strategies for further increasing yields should revolve around making additional NADH available.

## Background

Concerns about climate change and dwindling petroleum reserves are fuelling resurgence in the search for alternative, renewable fuels [1]. Among the possible candidates is hydrogen, and a great deal of active research is underway on hydrogen production, storage and utilization. Hydrogen is an attractive alternative fuel since it has the highest energy content per unit mass of any known fuel (143 GJ.t-1), it can easily be converted to electricity by fuel cells, and its combustion produces water as the only by-product. One requirement for a sustainable hydrogen economy is a renewable green technology for producing hydrogen. Biological hydrogen production could possibly be one such process that since it potentially uses renewable energy resources and operates at ambient temperature and atmospheric pressure [2,3]. Of the different possible approaches, dark fermentative hydrogen production has been the most extensively studied as it is viewed as being closer to near term application and uses readily available waste streams [4,5]. Hydrogen metabolism is widespread among microbes which have evolved a variety of metabolic networks and biosynthetic machinery to deal with this simple molecule. Many microorganisms can obtain energy by metabolically coupling hydrogen oxidation to a various kinds of electron acceptors such as fumarate, sulphate, carbon monoxide and oxygen [6]. Other organisms, carrying out fermentation in anaerobic environments, reduce protons to hydrogen as a means of disposing of excess reducing equivalents from oxidative metabolic pathways [7].

There are two major enzymes, [FeFe] and [NiFe] hydrogenases, containing complex active sites composed of metal ions and carbon monoxide and cyanide ligands, responsible for the production of hydrogen through the reduction of protons [8]. [NiFe] hydrogenase is widespread in both bacteria and archaea, has a broad range of substrate specificity, and has a relative low and reversible sensitivity to oxygen [9]. Unlike these hydrogenases, [FeFe] hydrogenases are limited to a narrow spectrum of bacteria and a few unicellular eukaryotes and they are extremely sensitive to irreversible oxygen inactivation [10]. [NiFe] hydrogenases consist of a large subunit, containing the Ni-Fe catalytic centre, and a small subunit, which has three Fe-S clusters which transfer electrons from the external electron donor to Ni-Fe active centre to reduce the protons. The molecular assembly of [NiFe] hydrogenase necessary to make it biologically active and functional requires the concerted actions of several maturation systems [11,12].

Although dark hydrogen fermentation is attractive, there are a number of challenges to its implementation. The major bottleneck is the low yields that are obtained; at most 2 mol H_2_ / mol glucose for enteric bacteria, such as *Escherichia coli*, or 4 mol H_2_ / mol glucose for strict anaerobes carrying out *Clostridial* type fermentations [2,4,7]. Various attempts have previously been made to improve biological hydrogen production in terms of molar hydrogen yields and cumulative hydrogen production rates [13-15]. *E. coli* has been the organism of choice for metabolic engineering given the ease of genetic manipulation in this organism and the large tool box that is available. Moreover, it is intrinsically of interest given its wide substrate specificity; it is capable of producing hydrogen from a variety of six and five carbon sugars and sugar derivatives [16]. Different approaches have been applied and it has been shown that it is possible to attain close to the theoretical yield for this organism of 2 H_2_ / mol glucose [17-19].

However, all metabolic engineering approaches using the native metabolic machinery are restricted to a maximum yield of 2 H_2_ / mol glucose since *E. coli* is normally unable to drive hydrogen production with NAD(P)H. Thus, deriving additional hydrogen from reduced electron carriers (NADH, NADPH) formed during substrate degradation requires development of metabolically engineered strains with the introduction and expression of non-native hydrogen producing pathways including foreign hydrogenases. Moreover, this approach has the potential for increasing other properties of interest including the use of protein engineering to channel electron flow, and to improve oxygen tolerance. Although there have been a number of previous attempts to introduce non-native hydrogen producing pathways into *E. coli*, in general the hydrogen production yields have been quite low. In this study, we have attempted to drive hydrogen evolution from NADH generated by cellular metabolism through the expression of the soluble, reversible, NAD-linked [NiFe] hydrogenase (SH-H_2_ase) operon from *Ralstonia eutropha* and have examined hydrogen yields in metabolically engineered non-hydrogen producing *E. coli* strains altered so as to potentially produce higher cellular levels of NADH.

## Results and discussion

### Rationale

**Figure 1:**
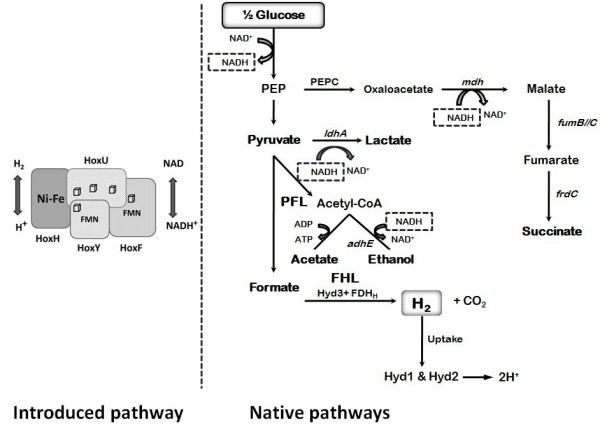
**Native and engineered metabolic pathways involved in hydrogen production by *E. coli.*** On the right is shown the main multiple pathways of mixed acid fermentation. Key enzymes and enzyme complexes are indicated by either the genetic nomenclature or the commonly used pathway abbreviation: Phosphoenolpyruvate (PEP); Phosphoenolpyruvate carboxylase (PEPC); Fumarate reductase (*frdC*); Lactate dehydrogenase (ldhA); Pyruvate formate lyase (PFL); Formate hydrogen lyase (FHL); Hydrogenase 3 (Hyd 3); formate dehydrogenase-H (FDHH); Uptake hydrogenases; hydrogenase 1 (Hyd 1) and hydrogenase 2 (Hyd 2); fumarase (*fumB*); fumurate reductase (*frdC*). Points where NADH is produced or consumed are noted. On the left is a schematic of the SH hydrogenase, which, if functional, might consume NADH, reducing protons to hydrogen.

In the absence of exogenous electron acceptors, anaerobically grown *E. coli* carries out a mixed acid type fermentation. Sugars are degraded to pyruvate by the glycolytic pathway, producing ATP and reducing NAD^+^ to NADH. The amount of NADH that is produced depends upon the redox state of the substrate, and this in turn controls fermentation product distribution. Pyruvate is mainly converted to formate and acetyl-CoA. Under the appropriate conditions, usually acidic pH, formate is broken down via the formate-hydrogen lyase pathway producing CO_2_ and H_2_. Thus, *E. coli* is only capable of the production of a maximum of 2 H_2_ per mole of glucose that enters glycolysis (Figure 1). The NADH that is generated during anaerobic growth on sugars must be oxidized to NAD^+^ for glycolytic metabolism to continue since NAD^+^ is a necessary cofactor for the oxidation of glyceraldehyde. Although in theory NADH could be oxidized by the reduction of pyruvate to lactate by lactate dehydrogenase, in practice this pathway is only fully expressed under acidic conditions, and does not seem to be sufficient on its own to permit anaerobic growth. Therefore, mutants deleted for alcohol dehydrogenase (*adhE*) cannot grow anaerobically on sugars more reduced than glucuronate [20]. Thus, *adhE* mutants might possess excess levels of NADH when incubated anaerobically. Some NADH can be recycled through the oxidation of oxaloacetate to malate, leading ultimately to the formation of succinate, but again, this side pathway is not sufficient in itself to permit anaerobic growth on sugars. Previous attempts to introduce novel hydrogen pathways had only given very low activities. Thus, the goal of the present research was to attempt to introduce a heterologous pathway that would allow the production of substantial amounts of hydrogen by reoxidizing NADH, thus potentially allowing for additional hydrogen production while, depending upon the strain, rescuing growth of some mutant strains incapable of anaerobic growth due to an inability to reoxidize sufficient amounts of NADH (Figure 1).

### Initial overexpression of SH hydrogenase

In order to examine the potential for engineering *E. coli* to produce hydrogen from NADH, we chose to express the SH hydrogenase from *Ralstonia eutropha* H16 (Table 1). The SH operon consists of nine genes; *hoxFUYHWI* and *hypA2B2F2*[21]. HoxHY is the hydrogenase module and HoxFU is a NADH dehydrogenase. hoxW encodes an highly specific endopeptidase required for the C-terminal processing of HoxH during hydrogenase maturation [22]. Although SH H_2_ase is usually isolated as a heterotetrameric protein (HoxHYFU), HoxI has been shown to provide a NADPH binding domain to a hexameric form of SH that can be isolated under certain conditions [23]. *hypA2B2F2* are duplicate copies of three of the seven R. eutropha hydrogenase maturation genes (*hyp*). Interestingly, they can substitute for *hypA1B1F1* in the maturation of both the SH hydrogenase and the MBH (membrane bound) hydrogenase [24]. A previous attempt to express the SH operon in *E. coli* from its native promoter (P_SH_) was unsuccessful [25], presumably because there is an absolute requirement for the transcriptional activator HoxA for expression from this promoter [21]. Therefore, we wished to express the SH operon from a promoter active in *E. coli*. Plasmid pJWPH5 for expression of the SH operon in *E. coli* under the control of the inducible *trc* promoter of the vector pTRC99A was constructed as described in Materials and Methods.

**Table 1:** Strains used

**Strain**	**Genotype**	**Reference/construction**
FTD147	Δ*hyaB* Δ*hybC* Δ*hycE*	Skibinski et al. 2002 [43]
JW135	Δ*hya-Km,* Δ*hyb-Km*	Menon et al. 1991 [26]
FTAB1	FTD147/ pJWPH5*	This study
FTAB4	FTD147, *ΔadhE, zch::Tn10*, pJWPH5	P1 DC1048 (*ΔadhE::Tn10*),
FTAB5	FTD147 *ΔarcA ::Tn10*, pJWPH5	P1 QC2575 (*ΔarcA::Tn10*),
FTDPH10	FTD147/pJWPH5 *adhE, ldhA*	P1 DC1048 (*ΔadhE::Tn10*), SE1752 (*ΔldhA::Tn10*)
DG2	FTD147/pJWPH5 *adhE, ldhA, arcA*	P1 DC1048 (*ΔadhE::Tn10*), SE1752 (*ΔldhA::Tn10*), QC2575 (*ΔarcA::Tn10*)
FTJWDC3	FTD147/pJWPH5 *adhE, mdh*	P1 DC1048 (*ΔadhE::Tn10*), JW3205-1 (*Δmdh::Tn5*)
FTGH2	FTD147/pJWPH5 *adhE, ldhA, mdh*	P1 DC1048 (*ΔadhE::Tn10*), SE1752 (*ΔldhA::Tn10*), JW3205-1 (*Δmdh::Tn5*)
DJ1	JW135/pJWPH5 *arcA*	P1 QC2575 (*ΔarcA::Tn10*)
JWGH1	JW135/pJWPH5 *adhE, ldhA, arcA*	P1 DC1048 (*ΔadhE::Tn10*), SE1752 (*ΔldhA::Tn10*), QC2575 (*ΔarcA::Tn10*)

^*^Plasmid containing the *Ralstonia eutropha* SH operon under the control of the *trc* promoter in pTrc99A.

The hydrogen evolution capacity of batch cultures of various strains of *E. coli* were tested (Table 2) under anaerobic conditions with LB medium as previously described [17]. *E. coli* possesses four hydrogenases; two of which, Hyd1 (*hya*) and Hyd2 (*hyb*), normally function in hydrogen oxidation, and two others, Hyd3 (*hyc*) and Hyd4 (*hyf*), which function physiologically in proton reduction. Hydrogen was evolved by strain BW535, wild-type for the four hydrogenases [26]. As expected, hydrogen was also evolved by strain JW135, which, being a Hyd1- Hyd2- (Δ*hya*-Km Δ*hyb*-Km) derivative of BW535, only lacks the hydrogenases that function to consume hydrogen. Strain FTD147, which lacks the hydrogen evolving Hyd3 as well as hydrogen consuming Hyd1 and Hyd2 [27], showed no hydrogen evolution (*E. coli* Hyd4 is inactive under these conditions [28]). The hydrogen evolution of strains containing pJWPH5 that had been altered so that they potentially produced more NADH was also tested. Derivatives of strain FTD147 (H_2_ase-) that lacked alcohol dehydrogenase (Δ*adhE*) or the aerobic/anaerobic regulator ArcA (Δ*arcA*), were examined for hydrogen evolution in anaerobically incubated LB-glucose (0.4%) medium (+ 0.05 mM IPTG) (Table 2). As discussed above, mutants lacking alcohol dehydrogenase should have excess NADH levels when incubated anaerobically and might consequently support hydrogen evolution by SH hydrogenase. However, there was no detectable hydrogen evolution by strain FTAB4 (FTD147/ pJWPH5 Δ*adhE*). Another strain, FTAB5, potentially able to produce increased levels of NADH under anaerobic conditions, was also examined. Strain FTAB5, which carries pJWPH5 (SH hydrogenase), is a Δ*arcA* derivative of strain FTD147. ArcA is a two-component regulator that is responsible for the anaerobic repression of synthesis of enzymes of the TCA cycle. Therefore, a strain mutated in ArcA might be expected to express the TCA cycle under anaerobic conditions, potentially permitting the generation of excess NADH from acetyl-CoA. Indeed, in vitro TCA cycle enzyme activities are greatly increased in ArcA mutants grown under anaerobic conditions, suggesting that additional NADH would become available if it could be more effectively oxidized under these conditions. However, there was no detectable hydrogen evolution by this strain either (Table 2).

**Table 2:** **Hydrogen evolution by various strains of *E. coli *incubated under anaerobic conditions**

***E. coli *strain**	**Relevant genotype**	**H**_**2**_
BW545	Wild type	+
JW135	Δ*hya-*Km Δ*hyb-*Km	+
FTD147	Δ*hyaB* Δ*hybC* Δ*hycE*	-
FTAB4	FTD147, Δ*adhE, zch::Tn10,* pJWPH5	-
FTAB5	FTD147 Δ*arcA ::Tn10*, pJWPH5	-

**Figure 2:**
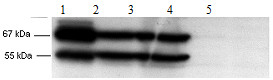
**Western blot analysis of expression of SH hydrogenase.** FTD147/pJWPH5) was cultured overnight at 30°C in LB medium with 0.05 mM IPTG under anaerobic conditions. The culture was harvested by centrifugation, sonicated, and centrifuged (15 min, 10,000 rpm). 25–35 μg pellet (lane 2) and supernatant (lane 3) were electrophoresed on 12% SDS-polyacrylamide gels (Laemmli and Favre (1973)), transferred to PVDF membrane, developed with primary anti-serum to SH hydrogenase, and revealed by chemiluminescence as previously described (Yakunin and Hallenbeck (1998)). A similarly cultured and prepared extract of FTD147 was also analyzed (supernatant, lane 4, pellet, lane 5) and found to be devoid of these protein bands. As a positive control, an aliquot of the supernatant of a 45 min 100,00 g centrifugation of a sonicated extract of *R. eutropha* H16 grown anaerobically overnight on NB medium at 30°C was also loaded (lane 1).

Given these results, we wished to verify that SH hydrogenase protein was present in these cells, even though no activity could be detected. Synthesis of SH hydrogenase proteins was checked by a Western blot (Figure 2) of an extract of strain FTD147/pJWPH5 grown under anaerobic conditions (LB + 0.05 mM IPTG). Prominent protein bands at 67 and 55 kDa were observed, corresponding to HoxF and HoxH respectively. Although there was a high level of expressed protein in the supernatant (Figure 2, lane 2), there appeared to be some inclusion body formation since a significant quantity was also recovered in the pellet obtained after centrifugation of the crude extract, produced by sonication, for 15 min at 10,000 rpm (Figure 2, lane 3). As a control, a high-speed supernatant of an extract of *R. eutropha* H16 grown anaerobically in FGN medium mineral salts medium [29] as previously modified [30] was included (Figure 2, lane 1). Thus, sufficient levels of SH hydrogenase appeared to be synthesized under anaerobic conditions in *E. coli* strain FTD147 strain carrying pJWPH5.

Lack of hydrogen evolution by anaerobically incubated FTD147-derivatives carrying pJWPH5 was therefore not due to lack of SH hydrogenase protein, but might rather be due to inadequate hydrogenase maturation. This was verified by checking the in vitro NAD-linked hydrogenase activity of extracts of anaerobically grown cultures of *R. eutropha* H16, *E. coli* FTD147, and *E. coli* FTAB1 (FTD147/pJWPH5) using a spectrophotometric assay [31]. As expected, high levels of H_2_-dependent NAD^+^ reduction was observed with the *R. eutropha* H16 extract, 5.87 ± 0.99 μmol NADH min^-1^ mg^-1^ and no reduction was seen with FTD147. However, only a very low level of activity was observed for FTAB1 (FTD147/pJWPH5), 0.39 ± 0.19 μmol NADH min^-1^ mg^-1^. Thus, it indeed appears that maturation of *R. eutropha* SH hydrogenase to a functional hydrogenase is very inefficient in *E. coli* grown anaerobically under these conditions.

### Anaerobic growth of *adhE* mutants carrying pJWPH5 (SH hydrogenase) on M9 glucose in the presence of nickel and iron

Since SH is a [NiFe] hydrogenase, we hypothesized that the low activity observed might be due to the insufficient supply of nickel and iron and therefore we assessed the effect of added nickel and iron on the formation of active SH hydrogenase [32]. First this was assayed using a growth test run under conditions such that only strains possessing an SH hydrogenase would grow. As described above, strains that are mutated in *adhE* have been reported to be impaired for growth under anaerobic conditions. We constructed various Δ*adhE* derivatives of both FTD147 and JW135 (FTGH2, FTJWDC3, JWGH1, DG2, FTDPH10) and verified that they were unable to grow under anaerobic conditions on sorbitol or glucose minimal media (not shown). Reasoning that growth could be restored if a means of reoxidizing NADH were introduced, these mutants were tested for the rescue of anaerobic growth on glucose by the introduction of pJWPH5, plasmid carrying the SH operon under the control of the *trc* promoter. Indeed, under these growth conditions, M9-glucose + IPTG + Ni + Fe, these derivatives were able to grow (not shown).

### NAD^+^ reduction in vitro

The growth results strongly suggested that SH was expressed and active in anaerobic cultures grown on nickel and iron supplemented M9-glucose. This was verified by assaying the SH hydrogenase activity of these strains. We measured the capacity of extracts to reduce NAD^+^ under a hydrogen atmosphere, i.e. to carry out hydrogen oxidation (Table 3). As expected, the positive control, an extract of *R. eutropha* H16 showed significant NAD^+^ reduction activity whereas the two *E. coli* strains, FTD147 and JW135, gave insignificant levels of activity. However, extracts of strains carrying pJWPH5 (SH hydrogenase) all showed varying but significant levels of SH hydrogenase activity in vitro. The parental strains, FTD147 and JW135 had specific activities close to 1 μmol NADH /min /mg protein, or 16% of that of an extract of *R. eutropha* H16. Extracts of strains carrying mutations that could be thought to increase cellular NADH levels, FTGH2, FTJWDC3, JWGH1, DG2, FTDPH10, and DG1, gave even higher specific SH hydrogenase levels, varying from 3.5 ± 0.1 μmol NADH /min /mg protein to 7.1 ± 0.31 μmol NADH /min /mg protein. These results (Table 3), obtained with nickel and iron amended M9, demonstrate the importance of medium supplementation with the metals required for cofactor synthesis since, in their absence, very little in vitro activity can be demonstrated.

**Table 3:** **In vitro NAD**^+^ reduction activity of various strains

**Strain**		**Relevant genotype**	**μmol NADH min**^**-1 **^**mg**^**-1**^
*R. eutropha* H16		Wild type	6.1 ± 0.4
FTD147		Δ*hyaB* Δ*hybC* Δ*hycE*	0
JW135		Δ*hya-Km,* Δ*hyb-Km*	0.04 ± 0.009
FTD147	+pJWPH5	Δ*hyaB* Δ*hybC* Δ*hycE*	0.94 ± 0.16
JW135	+pJWPH5	Δ*hya-Km,* Δ*hyb-Km*	1.09 ± 0..003
FTGH2	+pJWPH5	FTD147 *adhE, ldhA, mdh*	3.9 ± 0.06
FTJWDC3	+pJWPH5	FTD147 *adhE, mdh*	6.4 ± 0.10
JWGH1	+pJWPH5	JW135 *adhE, ldhA, arcA*	7.1 ± 0.31
DJ1	+pJWPH5	JW135 *arcA*	4.45 ± 0.003
DG2	+pJWPH5	FTD147 *adhE, ldhA, arcA*	3.56 ± 0.11
FTDPH10	+pJWPH5	FTD147 *adhE, ldhA*	3.5 ± 0.1

The *in vitro* NAD-linked hydrogenase activity of extracts of anaerobically grown cultures of *R. eutropha* H16, *E. coli* FTD147, and *E. coli* FTAB1 were assayed spectrophotometrically (Schneider and Schlegel (1976)). Twenty μg of extract were incubated in a stoppered cuvette containing 1.9 ml of hydrogen-saturated Tris buffer (50 mM, pH 8, 30C°) that had been flushed with hydrogen. The reaction was initiated by the addition of NAD^+^ to 0.8 mM, and the reduction of NAD^+^ followed at 365 nm.

The highest activities were observed with JWGH1, a JW135 (H_2_ase 3+) derivative carrying a mutation in the NADH consuming enzyme lactate dehydrogenase and in ArcA in addition to alcohol dehydrogenase, and FTJWDC3, a FTD147 (H_2_ase-) derivative mutated in malate dehydrogenase in addition to alcohol dehydrogenase. Somewhat lower levels of in vitro activity were observed with extracts of DJ1, a JW135 derived strain additionally mutated in *arcA*, and FTGH2, a FTD147 derivative mutated in both lactate dehydrogenase and malate dehydrogenase. A FTD147 derivative, DG2, carrying the same mutations as the JW135 derived strain JWGH1, gave only about 50% of the in vitro activity of that strain. Finally, FTDPH10, mutated in alcohol dehydrogenase and lactate dehydrogenase, gave only 50% of the highest observed in vitro activity, but even so this was more than three-fold higher than the parental strain, FTD147 carrying pJWPH5. It is clear from these results that, even though transcription is under control of the IPTG inducible *trc* promoter, higher levels of SH hydrogenase, as measured by in vitro activity, were present in strains in which the ability to reoxidize NADH anaerobically was compromised. The exact mechanism behind this enhancement is unclear, but might be related to general effects on growth. In addition, the results shown in Table 4 suggest that the effect of the introduction of multiple mutations in pathways that oxidize NADH appears to be additive, with abolition of malate dehydrogenase being more effective in a *adhE* strain than eliminating lactate dehydrogenase activity. At any rate, these results demonstrate the successful heterologous expression in *E. coli* of *R. eutropha* SH hydrogenase, multi-subunit [NiFe] hydrogenase capable of interacting with NAD+/NADH.

**Table 4:** In vivo hydrogen yields of strains expressing SH hydrogenase

**Strain**			**Yield**^**a**^
	**Background**	**Mutations**	
FTDPH10	FTD147 (Δ*hyaB* Δ*hybC* Δ*hycE*) /pJWPH5	*adhE, ldhA*	1.41 ±0.017
DG2	FTD147 (Δ*hyaB* Δ*hybC* Δ*hycE*) /pJWPH5	*adhE, ldhA, arcA*	1.46 ±0.015
FTJWDC3	FTD147 (Δ*hyaB* Δ*hybC* Δ*hycE*) /pJWPH5	*adhE, mdh*	2.08 ±0.016
FTGH2	FTD147 (Δ*hyaB* Δ*hybC* Δ*hycE*) /pJWPH5	*adhE, ldhA, mdh*	1.49 ±0.016
DJ1	JW135/pJWPH5	*arcA*	1.55 ±0.018
JWGH1	JW135/pJWPH5	*adhE, ldhA, arcA*	2.11 ±0.014

^a^mol H_2_/mol glucose consumed.

### *In vivo* hydrogen production by *E. coli* strains expressing SH hydrogenase

**Figure 3:**
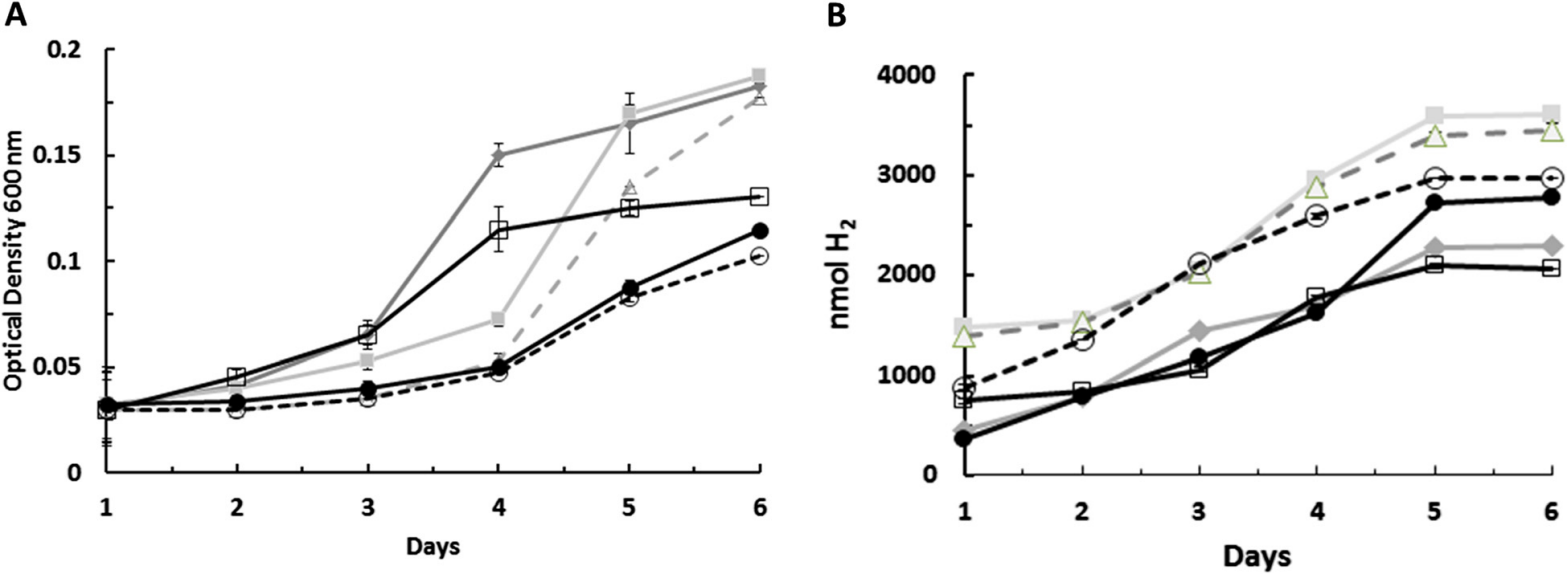
**Growth and *in vivo *hydrogen production by strains expressing SH hydrogenase.** Cultures, pregrown under the same conditions, were incubated at 37°C in anaerobic vials containing modified M9 glucose (+IPTG, Ni and Fe). Samples were taken periodically to measure OD **(A)** and hydrogen **(B)**. FTGH2/pJWPH5 (-♦-); FTJWDC3/pJWPH5 (-■-); JWGH1/pJWPH5 (-△-); DJ1/pJWPH5 (-○-); DG2/ pJWPH5 (-□-); FTDPH10/ pJWPH5 (-●-).

The results of the in vitro activity assays and the growth studies both provided evidence for the active expression of SH hydrogenase carried by pJWPH5. Therefore it was of interest to determine if these strains could produce hydrogen in vivo from glucose, demonstrating the establishment of a non-native hydrogen producing pathway in *E. coli*. The different strains were incubated anaerobically in modified M9-glucose. Growth was followed by measuring the OD (600 nm) (Figure 3A) and the hydrogen produced was assayed using gas chromatography (Figure 3B). All strains showed significant growth over the experimental period after a variable lag period (Figure 3A). Growth was highest, and at nearly the same level, in strains FTGH2, FTJWDC3, and JWGH1. FTGH2 and FTJWDC3 both carry *adhE* and *mdh* and FTGH2 *adhE*, *mdh* and *ldhA*. Final optical densities were appreciably lower in strains DJ1, DG2, and FTDPH10. Nevertheless, the growth of strains carrying *adhE*; FTGH2, FTJWDC3, JWGH1, DG2, and FTDPH10, demonstrates that they were capable of sufficient NADH reoxidation to permit growth. Since growth was only observed in strains carrying pJWPH5, NADH oxidation must have been provided by the action of SH hydrogenase.

Hydrogen production by these cultures was also examined (Figure 3B). All strains tested showed appreciable hydrogen evolution activity with final hydrogen levels of between 2.0 and 3.6 μmol H_2_ per vial (2 ml of culture). Strains FTJWDC3 (FTD147/pJWPH5 *adhE*, *mdh*) and JWGH1 (JW135/pJWPH5 *adhE, ldhA, arcA*) produced the greatest amount of hydrogen, whereas strains FTGH2 (FTD147/pJWPH5 *adhE, ldhA, mdh*) and DG2 (FTD147/pJWPH5 *adhE, ldhA, arcA*) produced the least. Interestingly, the two best hydrogen producers were also the strains that were shown to have the highest levels of SH hydrogenase activity in vitro (Table 3). Strain DG2, which gave one of the lowest SH hydrogenase activities in vitro, also produced the least amount of hydrogen. Taken together this suggests that hydrogen production levels are controlled by the amount of active SH hydrogenase that is present, but further work would be required to firmly establish this point. In addition, alterations in carbon flux through the different metabolic pathways operating in the various strains may have an influence as well. This could be determined my measurements of all the carbon fluxes involved, but such a study is beyond the scope of the present manuscript.

To measure the efficiency of hydrogen production, the amount of glucose consumed at the end point was determined and used to calculate the hydrogen yields, mol H_2_ produced / mol glucose consumed, of the different cultures (Table 4). All strains showed very good hydrogen yields, varying from 1.41 to 2.1 mol H_2_ / mol glucose, with strains FTJWDC3 and JWGH1 being the most efficient. Interestingly, since hydrogen yield and total hydrogen production are not always correlated, these were also the strains that produced the greatest amount of hydrogen. The yields observed here are higher than those normally observed with wild type cultures and are as high, or slightly higher, than the theoretical maximum for *E. coli* (see Figure 1 and earlier discussion). Of course, without any other metabolic changes, the maximum amount of hydrogen that could be produced by the SH hydrogenase would appear to be 2 H_2_/ glucose since two NADH are formed during glycolysis of glucose. However, strains that also contain the native H_2_ase 3 could in theory surpass this by producing in addition hydrogen from formate. Another fashion to exceed the 2 H_2_ / glucose limit would be to produce extra NADH by oxidizing some of the pyruvate through the TCA cycle.

These yields are also much higher than those obtained in previous studies where heterologous hydrogen producing pathways were introduced into *E. coli*. In several previous studies, ferredoxin-dependent [FeFe] hydrogenase pathways were introduced along with the enzymes necessary to reduce ferredoxin with either NADH or NADPH. However, yields were disappointingly low; 0.025 [33], 0.04 [34], 0.05 [35] mol H_2_ / mol glucose. On the other hand, when a [FeFe] hydrogenase was coupled with metabolism by the expression of a pyruvate:ferredoxin oxidoreductase, yields as high as 1.46 [36] mol H_2_ / mol glucose were obtained. Here we have introduced a [NiFe] hydrogenase dependent pathway and shown that it is capable of higher (44% greater than the highest previously reported) yields than the previously characterized [FeFe] hydrogenase dependent pathways. Others have previously reported the heterologous expression of [NiFe] hydrogenases [32,37-41], but only in two reports on the heterologous expression of the cyanobacterial *Synechocystis* [NiFe] hydrogenase were the in vivo hydrogen yields reported [39,41]. In one report in which a *E coli* strain devoid of native hydrogenases was used, a total hydrogen production of only 20 μmol H_2_ / liter was observed with a yield of only 0.0004 mol H_2_/ mol glucose [39]. The results of the present study represent a nearly 100 and 500 fold improvement respectively. In another study, the cyanobacterial [NiFe] hydrogenase was expressed in an *E. coli* strain which also possessed a native hydrogenase 3. Thus the two hydrogenase activities are confounded and it is difficult to determine exactly how much was due to the introduced hydrogenase, which might very well have had an indirect effect since its expression increased formate flux through H_2_ase 3 (Hyd3). One estimate, derived from the difference of the native strain and the recombinant strain, is that the heterologous hydrogenase contributed 0.67 mol H_2_ / mol of glucose, but again this is an overestimate since the primary effect seemed to be to drive additional hydrogen production from formate by the native H_2_ase 3 [41]. Here we have unequivocally shown that heterologous expression of the [NiFe] SH hydrogenase can give up to 2 mol H_2_ / mol glucose since we used a strain devoid of native hydrogenase activity. It might be thought that this amount of hydrogen from NADH is thermodynamically impossible, but a simple calculation using the Nernst equation shows that at 0.011 atm H_2_ (3.6 μmoles H_2_ in a head space of 8.3 ml) the equilibrium hydrogen redox potential would be −0.361 mV, within the range of the most recently determined equilibrium redox potential of the NAD/NADH couple, -0.378 mV [42]. Thus, an unusual NADH/NAD within the cell does not need to be invoked to explain the level of hydrogen production that we obtained in the present study. What the ultimate thermodynamic limits to hydrogen production are remains to be determined. Among other things, the applicability of a NADH/NAD determined for the whole cell to a specific biochemical reaction that may well be compartmentalized, or which may be proceeding under non-equilibrium conditions, remains to be demonstrated.

## Conclusion

The work reported here shows convincingly that a pyridine nucleotide dependent [NiFe] hydrogenase can be heterologously expressed in *E. coli* and produce large amounts of hydrogen from NAD(P)H produced by cellular metabolism. Hydrogen production is at least 50 fold greater than previously reported [39,41] This represents a significant advance in the ability to engineer hydrogen producing pathways in *E. coli*. Moving forward, a number of improvements could be made. Increasing flux through the system would be required to increase the rates of hydrogen production. In addition, a practical hydrogen production system would require that greater yields be obtained from the substrate, which could be brought about in several different ways. For one thing, more efficient coupling with the native hydrogen producing system, which produces hydrogen indirectly from pyruvate through the pyruvate:formate lyase system, should further increase yields. Another possibility would be to introduce a mechanism whereby additional NADH could be generated through the further metabolism of pyruvate, for example, through the anaerobic functioning of the citric acid cycle.

## Materials and methods

### Design and construction of expression system

Plasmid pJWPH5 for expression of the SH operon in *E. coli* under the control of the inducible trc promoter of the vector pTRC99A was constructed as follows. A 2.6 kb fragment of the 5′ end of the SH operon contained in plasmid pCH455 was PCR amplified using a primer which introduced an upstream XbaI site, and cloned into the XbaI/BamHI sites of pBluescript, giving pAB3. The SH operon was reconstituted by ligating BamHI-HindIII digested pCH455 and pAB3, giving plasmid pAB13. Finally, digestion of pAB13 with XbaI – HindIII gave a 14.2 kb fragment containing the SH operon, minus promoter sequence, which was cloned into pTRC99A, giving pJWPH5. Constructions were verified by restriction digests and PCR reactions.

### Preparation of metabolically engineered E.coli strains

*Escherichia coli* strains JW135 and FTD147 were used as the host strains for metabolic pathway alterations. FTD147 lacks the hydrogen evolving Hyd3 as well as hydrogen consuming Hyd1 and Hyd2 [27], and shows no hydrogen evolution under the conditions employed in this study (*E. coli* Hyd4 is inactive under these conditions [28]). Various metabolic alterations were made that would potentially increase NADH levels and thus provide substrate for the SH hydrogenase. Thus, different NADH utilizing pathways were blocked by mutating; *adhE*, *ldhA*, and/or *mdh*. Mutations were introduced into the parental strains P1 bacteriophage transduction (DC1048-Δ*adhE*::Tn10 TcR, SE1752-Δ*ldhA*::Tn10 TcR, JW3205-1-*Δmdh*::Tn10 kanR, QC2575-*ΔarcA*::TcR). Mutants are typically designated as *collie. coli* FTGH2 (Δ*ldhA*, Δ*adhE*, Δ*mdh*); FTJWDC3 (Δ*adhE*, Δ*mdh*); DG2 (Δ*ldhA*, Δ*adhE*, Δ*arcA*), FTDPH10 (Δ*ldhA*, Δ*adhE*) respectively. To make strains carrying multiple mutations, the tet resistance marker was removed by growing them on Maloy Nunn medium [43]. Phenotypes were confirmed by high performance liquid chromatography and growth was scored on minimal M9-Sorbitol medium under anaerobic conditions (Additional file 1: Figure S1). pJWPH5 carrying the SH operon was introduced into the various strains by chemical transformation.

### Heterologous expression of SH in metabolically engineered *E.coli* strains

All *E. coli* strains were grown overnight aerobically at 37°C in 5 ml LB medium with the appropriate antibiotics; ampicillin (100 μg/ml), tetracycline (15 μg/ml), and kanamycin (25 μg/ml). Antibiotic concentrations were used at half their standard concentrations in M9 (1X) minimal medium. Preinocula were prepared by growing the mutants on modified M9-glucose medium containing; ampicillin (50 μg/ml), 100 μM FeCl_3_, 25 μM NiSO_4_ and 0.05 mM IPTG under anaerobic conditions. Hydrogen production assays were carried out by inoculating the same medium contained in anaerobic tubes sealed with butyl rubber stoppers and incubating under anaerobic condition with glucose as sole carbon source (0.4% w/v and 0.2% w/v).

### Analytical methods

The concentration of hydrogen in the collected gas was determined using a gas chromatograph (Shimadzu GC-8A) equipped with a thermal conductivity detector, a 1 m column packed with molecular sieve 5A and with argon as carrier gas. Bacterial growth was determined by measuring the optical density at 600 nm using a double beam spectrophotometer (Shimadzu). Glucose concentrations were determined spectrophotometrically at 490 nm using a phenol-sulphuric acid assay [44].

## Abbreviations

adhE: Gene encoding alcohol dehydrogenase; H2ase: Hydrogenase; Hyd1-4: Various [NiFe] hydrogenases of *E. coli*; hyp: [NiFe] hydrogenase maturation genes; IPTG: Isopropyl β-D-1-thiogalactopyranoside; ldhA: Lactate dehydrogenase; MBH: Membranse bound [NiFe] hydrogenase of *R. eutropha*; mdh: Malate dehydrogenase; rpm: Revolutions per minute; SH hydrogenase: Soluble [NiFe] hydrogenase of *R. eutropha*.

## Competing interests

The authors declare that they have no competing interests.

## Authors’ contributions

The manuscript was written through contributions of all authors. AB did the cloning and some of the original characterization. DG created the *E. coli* mutant strains and did final characterization. PCH carried out the experimental design, helped analyse results and wrote the manuscript. All authors read and approved the final manuscript.

## Authors’ information

AB is currently at:

Bionest, 55, 12e Rue, C.P. 10070 Grand-Mère, Québec G9B 6R7 Canada.

## Supplementary Material

Additional file 1: Figure S1 *adhE* strains are unable to grow anaerobically on reduced sugars. The constructed strains, DG2 and FTGH2 were tested for anaerobic growth on reduced sugars by streaking M9-sorbitol (supplemented with Ni, Fe and IPTG) and incubating at 37°C in anaerobic jars (left). As a positive control, these strains were shown to grow on the same medium incubated aerobically (Right). Click here for file
