# Supplementary material for: Increasing the metabolic capacity of Escherichia coli for hydrogen production through heterologous expression of the Ralstonia eutropha SH operon
Source: Biotechnol Biofuels. 2013 Aug 26;6:122. doi: 10.1186/1754-6834-6-122 (PMC3765991; doi:10.1186/1754-6834-6-122)
Supplement: Additional file 1: Figure S1 — adhE strains are unable to grow anaerobically on reduced sugars. The constructed strains, DG2 and FTGH2 were tested for anaerobic growth on reduced sugars by streaking M9-sorbitol (supplemented with Ni, Fe and IPTG) and incubating at 37°C in anaerobic jars (left). As a positive control, these strains were shown to grow on the same medium incubated aerobically (Right). [file 1754-6834-6-122-S1.docx]

**
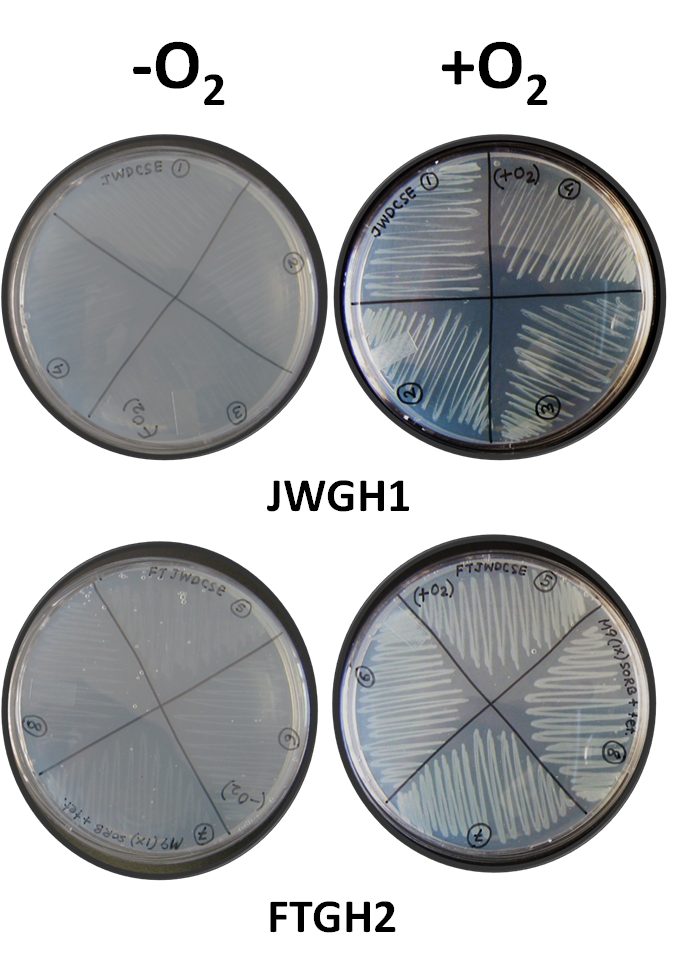
**

**Figure S1 *adhE* strains are unable to grow anaerobically on reduced sugars**

The constructed strains, DG2 and FTGH2 were tested for anaerobic growth on reduced sugars by streaking M9-sorbitol (supplemented with Ni, Fe and IPTG) and incubating at 37º C in anaerobic jars (left). As a positive control, these strains were shown to grow on the same medium incubated aerobically (Right)
